# Analysis of antenatal care, intranatal care and postnatal care utilization: Findings from the 2017 Indonesian Demographic and Health Survey

**DOI:** 10.1371/journal.pone.0258340

**Published:** 2021-10-12

**Authors:** Mabda Novalia Istifa, Ferry Efendi, Erna Dwi Wahyuni, Kadar Ramadhan, Qorinah Estiningtyas Sakilah Adnani, Jiun-Yi Wang

**Affiliations:** 1 Faculty of Nursing, Universitas Airlangga, Surabaya, Indonesia; 2 Department of Midwifery, Poltekkes Kemenkes Palu, Palu, Indonesia; 3 Faculty of Medicine, Universitas Padjadjaran, Bandung, Indonesia; 4 Department of Healthcare Administration, College of Medical and Health Science, Asia University, Taichung, Taiwan; 5 Department of Medical Research, China Medical University Hospital, China Medical University, Taichung, Taiwan; University of North Carolina at Greensboro, UNITED STATES

## Abstract

**Background and objective:**

Maternal healthcare utilization by young women and adolescent girls is associated with maternal health outcomes and plays a critical role in reducing maternal mortality rates in low- and middle-income countries. This study sought to analyze current data on antenatal care (ANC), intranatal care (INC), and postnatal care (PNC) utilization with a focus on mothers aged 15–24 years in Indonesia.

**Methods:**

This study was a secondary analysis of data from the 2017 Indonesian Demographic and Health Survey. The unit data analyzed 2,584 mothers aged 15–24 years who had delivered babies within the five-year period preceding the survey. Bivariate analysis and multiple logistic regression utilizing descriptive statistics were used to explore correlations between the independent variables and ANC, INC, and PNC visits.

**Results:**

Among the mothers included in the study, the prevalence of service utilization was 90.9% for ANC, 79.4% for INC, and 68.9% for PNC. Women’s age, education level, number and birth order of children, difference in age between the mother and her husband, her husband’s occupation, wealth index, access to the health service, and regional factors were significantly associated with the utilization of ANC, INC, and PNC services.

**Conclusion:**

This study provides insights for policymakers on how to strengthen healthcare policies and laws with the aim to improve maternal healthcare services for mothers aged 15–24 years. To improve maternal healthcare utilization among young mothers, national policy should focus on service equality, accessibility, and reliable implementation.

## Introduction

In 2017, the World Health Organization (WHO) estimated that the global maternal mortality rate was 211 per 100,000 live births [1]. The United Nations (UN) set a Sustainable Development Goals target to reduce the maternal mortality rate to less than 70 per 100,000 live births, with no individual country exceeding 140 maternal deaths per 100,000 by 2030, but meeting this goal is a difficult challenge [2], particularly for Indonesia, where the maternal mortality rate was three times higher than that set for the UN Millennium Development Goals (MDGs) in 2015 [3]. Indonesia is an lower-middle-income country with approximately 265 million inhabitants and is located in Southeast Asia. For many decades, Indonesia has put forth significant effort to reduce the mortality rate through maternal healthcare programs. Although the maternal mortality rate declined from 390 per 100,000 live births in 1991 to 305 in 2015, Indonesia continues to contribute to the high global maternal mortality rate [1, 4, 5], and its rate is higher than other Southeast Asian countries. Following the UN MDGs target of 102 per 100,000 live births set in 2015 [1, 4], Indonesia set a new target in 2020 to reduce maternal mortality rates to 131 per 100,000 live births by 2030 [6], which will require a 43% reduction in less than ten years is required to reach the new critical goal. This ambitious target seems too difficult to achieve, as Indonesia failed to reach the target set in the UN MDGs in 2015.

Maternal health outcomes are among the country’s fundamental health system performance indicators to reach the target set in the UN’s Sustainable Development Goals. Optimum maternal healthcare utilization, such as antenatal care (ANC) visits, skilled birth attendants or intranatal care (INC), and postnatal care (PNC) services are associated with maternal health outcomes. The term “skilled birth attendants” refers to the international agreement regarding “skilled health personnel (competent healthcare professionals) providing care during childbirth” [7–9]. However, a lack of maternal healthcare utilization frequently occurs [10, 11], primarily in low- and middle-income countries, including Indonesia [7, 11, 12].

Most maternal mortality deaths are either treatable or preventable, which indicates that maternal mortality among women could be avoided if critical interventions during ANC, INC, and PNC are undertaken. Most causes of maternal mortality—ranging from severe bleeding to eclampsia and sepsis—could be prevented by providing appropriate maternal healthcare with high-quality maternity services [13]. In the Indonesian setting, making ANC, INC, and PNC services available and accessible are a critical strategy to reduce the high rate of maternal mortality deaths. This follows the WHO’s guideline in 2016, which state that a minimum of eight ANC visits is associated with better quality of life throughout pregnancy and a positive birth experience [14]. The Ministry of Health of Indonesia 2018 strategic plan, a new maternal health program, Expanding Maternal and Neonatal Survival, focuses on improving maternal health care and aims to ensure that every woman has access to quality maternal health care, including childbirth assistance by skilled health personnel in healthcare facilities, with a target of 85% of pregnant women attending a minimum of four ANC (80%) and PNC (90%) visits. A recent publication in Indonesia revealed that 80%, 75%, and 66% of pregnant women aged 15–24 years visited ANC, INC, and PNC service providers, respectively, which is lower than the national target. The national target to improve maternal health outcomes for mothers aged 15–49 years determined that 90% of pregnant women will complete at least four ANC visits, 82% of deliveries will be assisted by skilled birth attendants, and 85% of mothers will attend PNC visits [4]. Compared to other age groups, mothers aged 15–24 years had the lowest percentage of visits to maternal healthcare services, with a gap of approximately 10% [4, 15].

Pregnancy among adolescents aged 10–19 years contributes significantly to maternal mortality rates worldwide [16]. In 2018, 650 million girls were married at younger than 20 years old, and approximately 21 million girls aged 15–19 years were pregnant. It was estimated that each year, there are 10 million unintended pregnancies among adolescents and 44 births for every 1,000 pregnancies [16, 17]. The national report revealed that the percentage of early marriage among adolescents in Indonesia was 23%, while the birth rate among adolescent girls and young women was 69 per 1,000 births in rural areas and 32 per 1,000 births in urban areas. With approximately 44 million adolescent girls, attention should be paid to their utilization of maternal healthcare services [18, 19]. Previous studies have shown that low maternal healthcare utilization among young mothers was influenced by several factors, including sociodemographic factors—such as age, educational status, employment, number of children, the wealth index, and media access [20]. The studies demonstrated that autonomy among young women was associated with maternal healthcare utilization [21]. In the Indonesian context, few studies have been conducted to analyze adolescent girls’ needs in maternal healthcare. The rationale for selecting the 15–24 age range was the high rate of newborn deaths among mothers in this group in Indonesia [4]. Thus, this study analyzed the utilization of ANC, INC, and PNC by mothers in this age group in Indonesia, based on data reported by the 2017 Indonesian Demographic and Health Survey (IDHS) data. The results of this secondary analysis may shed light for policymakers in the challenge to develop strategies and policies regarding the utilization of ANC, INC, and PNC services among young women, with the ultimate aim to reduce the risk of preventable maternal mortality among adolescents in Indonesia.

## Materials and methods

### Design and data source

This study was a secondary data analysis utilizing the data from the 2017 IDHS, which was conducted as part of the international Demographic and Health Survey (DHS) program. The unit data analyzed 2,584 mothers aged 15–24 who had delivered babies within the five-year period preceding the survey. Ethical clearance for the IDHS 2017 data collection project was obtained from ICF International’s Institutional Review Boards and the National Review Board of the Ministry of Health of Republic of Indonesia. The survey ensured international ethical standards of confidentiality, anonymity, and informed consent, the latter of which was provided by all participants during the survey. The cross-sectional study represents 1,970 census blocks of urban and rural areas in Indonesia. The survey was conducted on both national and provincial data by employing a two-stage stratified cluster sampling method. The first stage involved the selection of several census blocks utilizing systematic sampling of proportional sizes. In the second stage, 25 ordinary households were selected from the list of households within the census blocks. The researchers obtained permission to utilize the unit data for this study via the DHS program. All identifying information was deleted from the data. This study used survey questionnaires conducted by ICF to provide up-to-date estimates of basic demographic and health indicators. Specifically, the IDHS study provides a broad overview of population-specific problems in Indonesia. Individual recoding data was used for this analysis, and weighting was applied to account for the complex sampling design. We applied weights to the women’s data during the analysis, as the unit of analysis was women.

### Variables

The dependent variables were ANC, skilled birth attendants or INC, and PNC. All dependent variables were selected following the recommendations of the Ministry of Health of Indonesia. In this study, ANC visits were categorized into two groups: (1) less than four visits and (2) four or more visits. INC and PNC were divided into two categories: (1) Yes and (2) No. INC was defined as yes if the delivery took place in a health facility and was attended by a skilled provider. Although the WHO’s new guidelines from 2016 recommended a minimum of eight ANC visits, we selected four times or more based on data provided from the 2017 IDHS conducted over the five-year period preceding the survey. PNC must be performed by a skilled provider a minimum of three times: between the first six hours to the third day after delivery, between the fourth and 28^th^ day after delivery, and between the 29^th^ and 42^nd^ day after delivery. The data was based on the mother’s perceptions of PNC visits during the postpartum period. In this study, independent variables related to socioeconomic and geographic factors, including age, education level, employment status, number of children, birth order, perception of access to healthcare facilities, mass media exposure, and place of residence. The mother’s age was divided into two categories: 15–19 years and 20–24 years. The education levels of the mothers and their husbands were grouped into primary, secondary, or higher education. The employment status of both the mothers and their husbands was divided into two categories: not working or working. The age difference between the mothers and their husbands was divided into two categories: wife older than husband and wife younger than husband. Number of children was categorized into no children and one to three children, while birth order was categorized into first birth, second birth, and third birth. Barriers to access to healthcare were categorized into big problem and not a problem. Exposure to any mass media—the percentage of respondents exposed to specific media on a weekly basis—was divided into no exposure and some exposure. Place of residence was categorized into rural and urban areas, while provinces were defined as East, Middle, or West Indonesia.

### Statistical analysis

Distributions of the sociodemographic characteristics, as well as ANC, INC, and PNC utilization, were represented using descriptive statistics. The chi-square test was used to explore associations between the independent variables and maternal healthcare utilization (ANC, INC, and PNC visits). Multiple logistic regression analysis was performed to analyze ANC, INC, and PNC utilization among mothers aged 15–24 years. Measurement of the associations among variables was performed as an odds ratio (OR) and at a 95% confidence interval (CI). Significant variables were expressed with a *p*-value and 95% CI. All statistical analyses were performed using Stata 16 software. A *p*-value of less than 0.05 was considered significant. The inclusion criteria—mothers aged 15–24 years who had received ANC, INC, and PNC in the five years preceding the survey—were obtained from IDHS, while the exclusion criteria were incomplete or unavailable data. The number excluded due to incomplete data was 544 observations.

## Results

### Characteristics of the respondents

Table 1 depicts the distribution of ANC, INC, and PNC utilization among mothers aged 15–24 years and respondents’ sociodemographic factors. Of the 2,584 mothers aged 15–24, 90.9% had attended four or more ANC visits, 79.4% had skilled birth attendants, 68.9% had attended PNC visits in Indonesia, 87.5% were in the 20- to 24-years-old age range, and 60.4% were currently not working. Nearly three-quarters of women had a secondary education, and almost all women had one to three children. Nearly all of the women were younger than their husbands. Almost all of the husbands were working, and more than half had a secondary education. Nearly all women were exposed to mass media, and 87.6% had no difficulty accessing maternal healthcare. The proportion of women living in rural areas (59.5%) was higher than those living in urban areas (40.5%), and 80.3% of the women resided in West Indonesia. Additional information about the respondents’ characteristics is available in Table 1.

**Table 1:** Characteristics of mothers aged 15–24 years in Indonesia.

Variable	N (n = 2584)	%
Antenatal Care		
Less than 4x	235	9.1
4x or more	2349	90.9
Intranatal Care		
No	532	20.6
Yes	2052	79.4
Postnatal Care		
No	804	31.1
Yes	1780	68.9
Age		
15–19 years	322	12.5
20–24 years	2262	87.5
Respondent’s Occupation		
Not working	1562	60.4
Working	1022	39.6
Respondent’s Education Level		
Primary	515	19.9
Secondary	1930	74.7
Higher	139	5.1
Respondent’s and Husband’s Age Difference		
Wife older than husband	190	7.4
Wife younger than husband	2394	92.6
Husband’s Occupation		
Not working	21	0.8
Working	2563	99.2
Husband’s Education Level		
Primary	693	26.8
Secondary	1713	66.3
Higher	178	6.9
Number of Children		
No surviving children	155	6.0
1–3 children	2429	94.0
Wealth Index		
Poorest	595	23.0
Poorer	692	26.8
Middle	626	24.2
Richer	443	17.2
Richest	228	8.8
Birth Order		
1st birth	2131	82.5
2^nd^–3^rd^ birth	453	17.5
Problems in Accessing Healthcare		
Big problem	319	12.4
Not a problem	2265	87.6
Exposure to Mass Media (newspaper/tv/radio)		
No exposure	49	1.9
Some exposure	2535	98.1
Resident		
Urban	1047	40.5
Rural	1537	59.5
Province		
East of Indonesia	69	2.7
Middle of Indonesia	439	17.0
West of Indonesia	2076	80.3

### Bivariate analysis

Table 2 presents detailed results of the bivariate analysis. It shows variables related to the women’s ANC, INC, and PNC utilization (*p* < 0.05), which includes their age, education level, birth order, age difference compared to husband’s age, husbands’ employment status and education level, number of children, wealth index, perception of access to healthcare facilities, exposure to mass media, and place of residence including province.

**Table 2:** Bivariate analysis of ANC, INC, and PNC utilization among mothers aged 15–24 years in Indonesia.

Variable	ANC					INC					PNC				
	less than 4x (n = 235)		4x or more (n = 2,349)		χ^2^	No (n = 532)		Yes (n = 2052)		χ^2^	No (n = 804)		Yes (n = 1780)		χ^2^
	n	%	n	%		n	%	n	%		n	%	n	%	
**Respondents’ sociodemographic factors**															
Age															
15–19 years	52	16.3	270	83.7	20.0***	87	26.9	236	73.1	8.6*	113	35.2	209	64.8	2.8
20–24 years	183	8.1	2079	91.9		445	19.7	1816	80.3		691	30.5	1571	69.5	
Occupation															
Not working	150	9.6	1412	90.4	1.1	342	21.9	1220	78.1	3.9	503	32.2	1058	67.8	2.2
Working	85	8.4	937	91.6		190	18.6	832	82.4		301	29.4	721	70.6	
Education level															
Primary	59	11.5	455	88.5	4.5	175	34.1	339	65.9	69.2***	175	34.1	339	65.9	3.2
Secondary	155	8.6	1764	91.4		334	17.3	1596	82.7		591	30.6	1339	69.4	
Higher	10	7.4	129	92.6		23	16.2	117	83.8		38	27.2	102	72.8	
Birth order															
1st birth	173	8.1	1958	91.9	13.3***	404	19.0	1727	81.0	19.0**	615	28.9	1516	71.1	28.2***
2nd–3rd birth	62	13.7	391	86.3		128	28.2	325	71.8		189	41.8	264	58.2	
**Husbands’ sociodemographic factors**															
Wife–husband age difference															
Wife older than husband	9	5.0	180	95.0	4.1*	31	16.2	159	83.8	2.3	53	27.9	137	72.1	0.9
Wife younger than husband	226	9.4	2168	90.6		4501	20.9	1893	79.1		751	31.4	1643	68.6	
Husband’s occupation															
Not working	7	32.3	14	67.6	13.2**	8	40.6	12	59.4	4.9	5	26.7	15	73.3	0.2
Working	229	8.9	2334	91.1		524	20.4	2040	79.6		799	31.2	1765	68.8	
Husband’s education level															
Primary	76	11.0	616	89.0	4.0	211	30.5	481	69.5	55.3***	241	34.8	452	65.2	5.8
Secondary	145	8.4	1568	91.6		291	17.0	1423	83.0		511	29.8	1202	70.2	
higher	14	8.0	164	92.0		30	16.9	148	83.1		52	29.2	126	70.8	
**Household factors**															
Number of children															
No surviving children	23	15.0	132	85.0	6.8*	44	28.4	111	71.6	5.9*	67	43.3	88	56.7	11.1*
1–3 children	212	8.7	2217	91.3		488	20.1	1941	79.9		737	30.3	1692	69.7	
Wealth index															
Poorest	96	16.0	500	84.0	50.3***	234	39.4	360	60.6	200.9***	215	36.2	379	63.8	18.1*
Poorer	63	9.2	628	90.8		154	22.3	538	77.7		230	33.7	462	66.7	
Middle	44	7.1	582	92.9		82	13.0	544	87.0		166	26.5	460	73.5	
Richer	22	5.1	421	94.9		48	10.8	396	89.2		120	27.0	334	73.0	
Richest	10	4.4	218	95.6		14	6.2	214	93.8		73	32.0	155	68.0	
Problems in accessing healthcare															
Big problem	56	17.5	264	82.5	30.2***	87	27.3	232	72.7	9.6*	123	38.6	196	61.4	9.1*
Not a big problem	179	7.9	2085	92.1		445	19.7	1820	80.3		681	30.1	1584	69.9	
Exposure to mass media (newspaper/tv/radio)															
No exposure	15	30.9	34	69.1	27.9***	18	35.9	32	64.1	7.0*	22	45.5	27	54.5	4.7
Some exposure	220	8.7	2315	91.3		514	20.3	2020	79.7		782	30.8	1753	69.2	
**Region**															
Resident															
Urban	67	6.4	980	93.6	15.0**	101	9.7	945	90.3	123.7***	334	32.0	712	68.0	0.6
Rural	168	10.9	1369	89.1		431	28.0	1107	72.0		470	30.5	1068	69.5	
Province															
East of Indonesia	19	28.3	49	71.7	30.8***	37	53.5	32	46.5	48.2***	27	39.9	41	60.1	2.5
Middle of Indonesia	41	9.3	399	90.7		100	22.8	339	77.2		138	31.4	301	68.6	
West of Indonesia	175	8.4	1901	91.6		395	19.0	1681	81.0		639	30.8	1438	69.2	

*p < 0.05

**p < 0.01

***p < 0.001.

ANC: antenatal care; INC: intranatal care; PNC: postnatal care.

### Multiple logistic regression analysis

Table 3 presents detailed results of the adjusted model of multiple logistic regression analysis. Mothers aged 20–24 have two times the odds of attending four and more ANC visits than mothers aged 15–19, adjusting for other sociodemographic characteristics (OR = 2.0; 95% CI = 1.3–3.2). Furthermore, when adjusting for other sociodemographic characteristics, women who graduated with a secondary education had 1.7 times the odds having a skilled birth attendant at the health facility they attended than those who graduated with a primary education (OR = 1.7; 95% CI = 1.3–2.3). Women in their first pregnancy had 1.8 times the odds of utilizing ANC (OR = 1.8; 95% CI = 1.2–2.9) or INC visits (OR = 1.5; 95% CI = 1.1–2.1), and 1.7 times the odds of utilizing PNC visits (OR = 1.7; 95% CI = 1.3–2.3) than those in their second to third pregnancy. Women who were older than their husbands had 2.0 times the odds of attending at least four ANC visits than those who were younger (OR = 2.0; 95% CI = 1.0–3.8). Women whose husbands worked had 4.6 times the odds of utilizing ANC visits (OR = 4.6; 95% CI = 1.3–16.3) and 2.8 times the odds of receiving INC than mothers whose husbands were unemployed (OR = 2.8; 95% CI = 1.0–8.1). Women who had one to three children had 1.8 times the odds of utilizing PNC services than women who had no children (OR = 1.8; 95% CI = 1.2–2.7). Women in the most affluent group had 2.5 times the odds of utilizing ANC visits (OR = 2.5; 95% CI = 1.1–5.8) and had 4.9 times the odds for INC (OR = 4.9; 95% CI = 2.4–10.1) than those in the lowest group. Women who did not report serious problems with accessibility to healthcare facilities as a constraint had 2.0 times the odds of utilizing ANC visits than those who did (OR = 2.0; 95% CI = 1.3–3.0). Those in urban areas had 2.5 times the odds of having INC than those in rural areas (OR = 2.5; 95% CI = 1.7–3.6). Women who were living in Middle Indonesia had 2.8 times the odds of attending four or more ANC visits and had 3.4 times the odds having a skilled birth attendant compared to those who were living in East Indonesia (OR = 2.8; 95% CI = 1.5–5.6 and OR = 3.4; 95% CI = 2.3–5.1). Women who were living in West Indonesia had 2.4 times the odds of attending four or more ANC visits and had 3.1 times the odds having a skilled birth attendant compared to those who were living in East Indonesia (OR = 2.4; 95% CI = 1.3–4.8 and OR = 3.1; 95% CI = 2.1–4.6).

**Table 3:** Adjusted multiple logistic regression analysis of ANC, INC, and PNC utilization among mothers aged 15–24 years in Indonesia.

Variable		ANC			INC			PNC	
	AOR	95% CI		AOR	95% CI		AOR	95% CI	
		Lower	Upper		Lower	Upper		Lower	Upper
**Respondents’ factors**									
Age									
15–19 years	1.0			1.0			1.0		
20–24 years	2.0**	1.3	3.2	1.3	0.9	1.9	1.3	0.9	1.8
Education level									
Primary	1.0			1.0			1.0		
Secondary	1.0	0.6	1.5	1.7***	1.3	2.3	1.0	0.7	1.3
Higher	0.9	0.4	2.7	1.6	0.9	2.9	1.1	0.6	1.8
Birth order									
1st birth	1.8**	1.2	2.9	1.5*	1.1	2.1	1.7***	1.3	2.3
2nd–3rd birth	1.0			1.0			1.0		
**Husbands’ sociodemographic factors**									
Wife–husband age difference									
Wife older than husband	2.0*	1.0	3.8	1.2	0.7	2.0	1.1	0.8	1.6
Wife younger than husband	1.0			1.0			1.0		
Husband’s occupation									
Not working	1.0			1.0			1.0		
Working	4.6*	1.3	16.3	2.8*	1.0	8.1	0.7	0.3	1.9
Husband’s education level									
Primary	1.0			1.0			1.0		
Secondary	1.0	0.7	1.5	1.3	1.0	1.7	1.2	0.9	1.5
Higher	1.0	0.4	2.4	1.0	0.6	1.8	1.2	0.8	1.9
**Household factors**									
Number of children									
No surviving children	1.0			1.0			1.0		
1–3 children	1.9	1.0	3.5	1.6	1.0	2.6	1.8**	1.2	2.7
Wealth index									
Poorest	1.0			1.0			1.0		
Poorer	1.4	0.9	2.2	1.7**	1.2	2.4	1.0	0.8	1.4
Middle	1.7	1.0	2.7	2.7***	1.7	4.1	1.4*	1.0	2.0
Richer	2.2*	1.1	4.2	2.7***	1.6	4.4	1.4*	1.0	2.0
Richest	2.5*	1.1	5.8	4.9***	2.4	10.1	1.1	0.7	1.7
Problems in accessing healthcare									
Big problem	1.0			1.0			1.0		
Not a big problem	2.0**	1.3	3.0	1.1	0.7	1.7	1.3	1.0	1.9
Exposure to mass media (newspaper/tv/radio)									
No exposure	1.0			1.0			1.0		
Some exposure	2.2	0.8	5.6	0.7	0.3	1.7	1.4	0.7	2.9
**Region**									
Resident									
Urban	1.3	0.9	1.9	2.5***	1.7	3.6	0.8	0.6	1.0
Rural	1.0			1.0			1.0		
Province									
East of Indonesia	1.0			1.0			1.0		
Middle of Indonesia	2.8**	1.5	5.6	3.4***	2.3	5.1	1.4	0.8	2.3
West of Indonesia	2.4**	1.3	4.8	3.1***	2.1	4.6	1.2	0.7	2.0

*p < 0.05

**p < 0.01

***p < 0.001

ANC: antenatal care; INC: intranatal care; PNC: postnatal care; AOR: adjusted odds ratio; 95% CI: 95% confidence interval.

## Discussion

This study analyzed ANC, INC, and PNC utilization among mothers aged 15–24 years in Indonesia. Overall, it found that 90.9% had attended four or more ANC visits, 79.4% had skilled birth attendants, and 68.9% had participated in PNC visits in Indonesia. The findings indicated that maternal healthcare utilization was significantly associated with specific factors of the pregnant mother, husband, household, and region. The results showed that women aged 20–24 had two times the odds of attending four or more ANC visits than women aged 15–19 years old. A possible reason could be that younger women tend to feel unprepared when becoming new mothers and dealing with new roles and physical changes during adolescent pregnancies. This might be due to the fact that younger women tend to have mixed feelings and emotional instability when carrying out their new role as a mother [22, 23]. For instance, younger women tend to have limitations in their decision-making capacity due to age maturity and lack of maternal healthcare experience [22, 23]. This finding is congruent with research in Zambia that found a correlation between older women and ANC visits [24]. Therefore, age maturity is an essential factor in making decisions regarding marriage and pregnancy. Becoming a new mother, especially at a young age, requires substantial attention and support from family and health workers in maternal healthcare facilities. In the Indonesian setting, in accordance with the 1974 Marriage Law, the minimum age for marriage is 16 for girls and 19 for boys [25]. However, the Marriage Law allows parents to marry their children at a younger age under the cultural law [25]. With more than 350 ethnicities in Indonesia, enforcement of the Marriage Law to protect children from child marriage was considered inconsistent [26].

Regarding education level, women who graduated with a secondary education had 1.7 times the odds of having a skilled birth attendant at the health facility they attended than those who graduated with a primary education. This finding was in line with previous studies that found a correlation between the level of a woman’s education and the level of experience of the person who assisted her childbirth [24, 27–29]. A possible reason could be that women with a secondary education have higher maternal health literacy that enables them to select better maternal healthcare facilities, as they are more likely to be exposed to maternal education. A higher educational background may provide women with adequate knowledge leading to critical thinking regarding maternal health.

This study revealed that women in their first pregnancy had 1.8 times the odds of utilizing ANC visits and 1.7 times the odds of utilizing PNC visits than those in their second or third pregnancy. Previous research also showed that birth order is associated with maternal healthcare utilization [29, 30]. A possible reason could be linked to awareness of the transition period of motherhood; notably, the first experience of having children could play a vital role in the choice to use maternal healthcare. Our findings revealed higher odds among older women (20–24) and those in their first pregnancy. A possible reason could be linked to higher attainment of information, thus resulting in greater knowledge of the benefits in utilizing maternal services. The present study also revealed that husbands’ factors were correlated with maternal healthcare utilization among mothers aged 15–24 years in Indonesia. Women who were older than their husbands had 2.0 times the odds of attending at least four or more ANC visits than younger women. This result is not consistent with a previous study that found that the age gap between women and their husbands was not correlated to maternal healthcare utilization [30]. A possible reason could be that women who were older than their husbands were frequently associated with greater autonomy and were therefore empowered with the choice to seek maternal healthcare. Furthermore, women whose husbands worked had 4.6 times the odds of utilizing ANC visits and 2.8 times the odds of having INC than mothers whose husbands were unemployed. Previous studies supported this statement; working husbands have the economic stability to offer the best option for their family’s maternal health [24]. This might be due to the fact that financial security poses no economic barrier to maternal healthcare utilization, which can influence households to be better prepared to welcome a new member of their family and meet their needs.

Moreover, household factors, including the number of children, wealth index, distance to the healthcare facilities, and mass media exposure, were associated with maternal healthcare utilization. The findings revealed that women who had one to three children had 1.8 times the odds of utilizing PNC services than women who had no children. This finding, which is consistent with studies in Sub-Saharan African countries and Ghana [20, 31], might be due to an awareness of the importance of better maternal preparation that women acquire during motherhood. Moreover, it has been well-documented that the wealth index is associated with maternal healthcare utilization [30, 32]. Our study found that women in the most affluent group had 2.5 times the odds of utilizing ANC visits and 4.9 times the odds of receiving INC than the poorest group. This might be due to the fact that those in the wealthiest group did not experience an economic burden in meeting the maternal needs of their family. Our findings also found that women who did not report distance to healthcare facilities as a constraint had 2.0 times the odds of attending ANC visits than those who did. Previous studies also demonstrated that families tend to choose the nearest health facilities for obtaining maternal and healthcare services [33]. A possible reason could be the consideration of transportation costs and accessibility to healthcare facilities. As a young family, they might consider that other household needs must be met.

In this study, the final variable related to maternal healthcare utilization was regional factors. We found that those who were living in urban areas had 2.5 times the odds of receiving INC than those in rural areas. Women who were living in Middle Indonesia had 2.8 times the odds of attending four or more ANC visits and 3.4 times the odds of having a skilled birth attendant compared to those who were living in West Indonesia. Empirical evidence reveals that socioeconomic development, including quality transportation, roads, and health facilities, was not equally distributed throughout Indonesia, as there were large discrepancies in the quality of healthcare facilities across Indonesia’s regions [34–36]. It is undeniable that a short distance, better roads, and public transportation can influence women in urban areas to attend more-accessible healthcare facilities than women in rural areas. Intertwined, complex factors—such as socioeconomic differences and regional disparities—may hinder women’s access to maternal healthcare and result in them being unable to attend the minimum number of ANC appointments, have a skilled birth attendant, and attend PNC appointments.

This study used the most recent data available for Indonesia, which included national and provincial representation with a high response rate (>95%). This study has some limitations. The cross-sectional method, which measured all variables in the same period, cannot explain the causality. Furthermore, the analysis for this study fully accounted for the available data from IDHS, which resulted in limited coverage predictor variables. In addition, the survey was based on the mother’s response (self-reported) two weeks preceding the study, allowing for the possibility of recall bias. Previous studies also revealed similar limitations regarding the bias of the maternal recall of ANC, skilled birth attendance, and PNC [32].

## Conclusions

This study analyzed the current ANC, INC, and PNC utilization among mothers aged 15–24 years old who were living in Indonesia, revealing that utilization of these was significantly associated with many factors, such as age, education level, number of children, birth order, age difference between the mothers and their husbands, husband’s occupation, wealth index, access to health services, and regional factors. The different prevalence of utilization of ANC, INC, and PNC services and wide disparities in various aspects among mothers aged 15–24 years in Indonesia highlighted the challenges that young women face in utilizing maternal healthcare services. This study provides some insights for policymakers to strengthen healthcare policy and laws regarding maternal healthcare services for mothers aged 15–24 years. The need for national policy focuses on service equality, accessibility, and reliable implementation to improve maternal healthcare utilization among young mothers. Large-scale promotion of the need for maternal healthcare aimed at young mothers may enable them to become well-informed on this topic. Adequate implementation of tailored programs and interventions to improve maternal healthcare utilization among young mothers should be of significant concern to professional healthcare workers and policymakers at all levels.
